# Comprehensive History of *CSP* Genes: Evolution, Phylogenetic Distribution and Functions

**DOI:** 10.3390/genes11040413

**Published:** 2020-04-10

**Authors:** Guoxia Liu, Ning Xuan, Balaji Rajashekar, Philippe Arnaud, Bernard Offmann, Jean-François Picimbon

**Affiliations:** 1 Biotechnology Research Center, Shandong Academy of Agricultural Sciences, Jinan 250100, China; girlgx@sina.com (G.L.); xuanning3205613@sina.com (N.X.); 2 Institute of Computer Science, University of Tartu, Tartu 50090, Estonia; balajior@gmail.com; 3 Protein Engineering and Functionality Unit, University of Nantes, 44322 Nantes, France; philippe.arnaud@univ-nantes.fr (P.A.); bernard.offmann@univ-nantes.fr (B.O.); 4 School of Bioengineering, Qilu University of Technology, Jinan 250353, China

**Keywords:** tandem duplication, RNA mutation, adaptive process, lipid transport, xenobiotic resistance, neuroplasticity

## Abstract

In this review we present the developmental, histological, evolutionary and functional properties of insect chemosensory proteins (CSPs) in insect species. CSPs are small globular proteins folded like a prism and notoriously known for their complex and arguably obscure function(s), particularly in pheromone olfaction. Here, we focus on direct functional consequences on protein function depending on duplication, expression and RNA editing. The result of our analysis is important for understanding the significance of RNA-editing on functionality of *CSP* genes, particularly in the brain tissue.

This report reviews duplication, expression, evolution and RNA editing of *CSP* genes for neofunctionalization in insecticide resistance and neuroplasticity, with a particular special interest in functional properties of insect chemosensory proteins (CSPs).

Noticing that this gene family exhibits signs of RNA editing, we speculate that they play a role in interacting with diverse compounds, including mainly xenobiotics, lipids and fatty acids of the linoleic acid pathways. We do not attempt to give justice to the eluding nature of this protein family, but we attempt to address all the known aspects of the *CSPs* from genomic organization to expression analysis, which are perhaps important signature motifs of the multifunction. Accordingly, we report about gene duplication, ubiquitous expression in the whole insect body, expression in response to the application of insecticide, and new phylogenetic analyses before formulating a theory on the role of the pleiotropic nature of this protein gene family, which might be particularly important in pathways of cellular metabolism that regulate not only the immune system and digestive tract, but also the peripheral nervous system and brain.

In this study, we describe the genomic organization, chromosomal localization and gene structure of *CSPs* in the *Apis*/*Nasonia* model and a comparative analysis with *Bombyx*, *Pediculus* and *Tribolium* genomes, from which first genetic data about *CSPs* have been obtained. The choice to direct CSP research towards hymenoptera, in particular behaviors of bees and wasps, resides in the differences in the sensitivity of these insects to pesticides, social molecular pathways and the evolution of insect societies, as well as the complexity of adaptation and learning capacity. While many bee species are endangered on the brink of extinction as a result of excessive use of pesticides of all sorts, challenging pollination, biodiversity and environmental fate, the bee brain remains an exceptional model to see how insects can learn to associate odors and colors in a similar way to humans. 

## 1. Introduction: The Family of Chemosensory Proteins (CSPs)

### 1.1. A Very Ancient Malleable Protein

Chemosensory proteins are a class of small (10–12 kDa) soluble proteins reported for the first time by Nomura et al. (1982) as an up-regulated factor in the regenerating legs of *Periplaneta americana* [1]. Soon enough, the same protein was identified in the antennae and legs from sexually mature adult cockroaches with some apparent differences between females and males, rather suggesting a “*chemodevol”* function for this protein, i.e., contributing both to tissue development and the recognition of sex-specific signals such as sex pheromones [2,3,4,5,6]. They fold into a flexible prism constituted of six alpha-helices, a hydrophobic inner side particularly suitable for the transport of long aliphatic chains and for specific conformational changes on ligand binding [7,8,9,10,11].

However, multifunction in CSPs, as coined by Picimbon (2003), mainly refers to the ubiquitous tissue-distribution of this protein family from non-sensory organs such as the gut and fat body in the internal abdominal area to the sensory structures of the antennae, palps and legs [12]. In the silkworm moth *Bombyx mori*, most CSPs are found to be co-expressed in the pheromone gland and to be up-regulated in several tissues following insecticide exposure [13,14]. Coincidentally, CSPs are found to be crucial transporters not only for molecules as diverse as fatty acid lipids such as linoleic acid, but also for insecticide xenobiotics of plant oil origin such as cinnamaldehyde, as recently described in the sweetpotato whitefly *Bemisia tabaci* [15]. Such patterns in tissue-distribution and such ligand diversity invite us to debate further about the complexity and/or multi-functional aspects of this more and more challenging protein family. 

**Figure 1:**
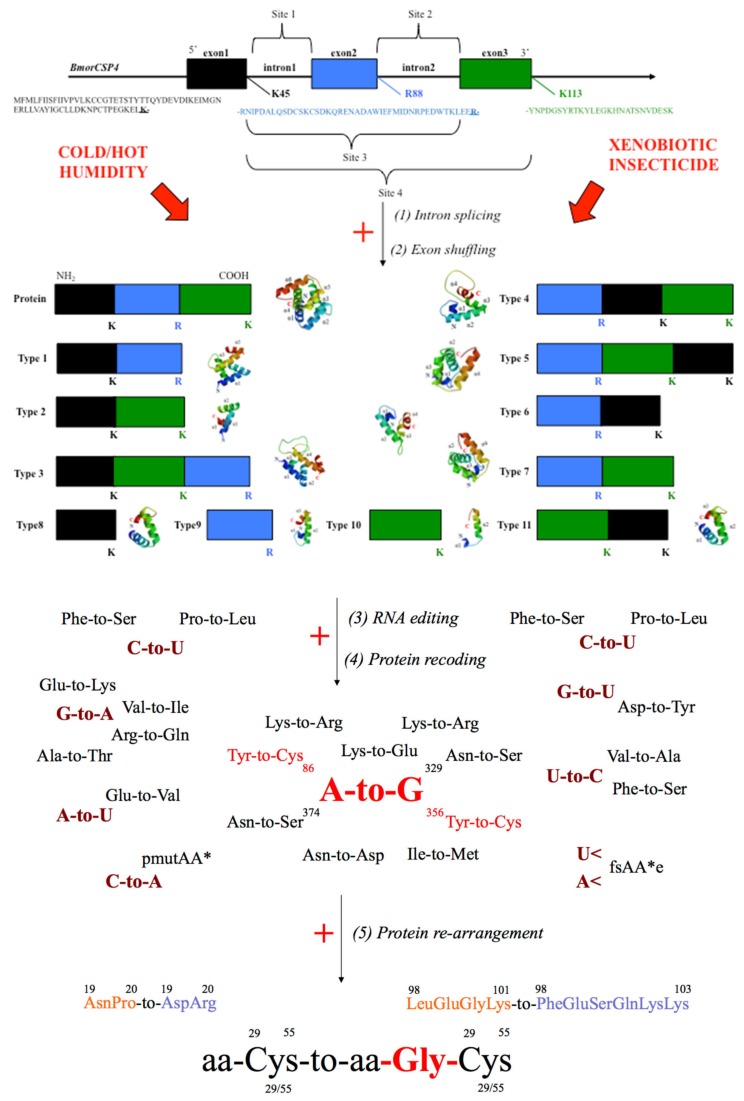
Gene splicing and RNA editing mechanism for diversification of chemosensory proteins (CSPs) under environmental change. An insect *CSP* gene structure such as lepidopteran *BmorCSP4* contains 2 introns and 3 exons [13,14]. (1) Genomic DNA (bold black line) is transcribed into premature mRNA yielding four possible sites for intron splicing. (2) The native protein sequence and eleven types of protein sequence variants can be produced by intron splicing, excision of non-coding regions (intron boundaries: K45, R88 and K113) and shuffling of coding regions (exon1 in black, exon2 in blue and exon 3 in green box). The folded shape of BmorCSP4 and a number of 11 new protein foldings (11 variants) can be generated from gene splicing. (3) The primary transcripts that are a faithful copy of the gene and variant mRNAs are all subject to further typo RNA editing, resulting in an increased number of genetic variants and protein subtypes [13,16,17,18,19,20]. Each mRNA is subject to mutations (A-to-G, A-to-U, C-to-A, C-to-U, G-to-A, G-to-U and/or U-to-C) depending on external conditions (cold/hot temperature, humidity and/or exposure to xenobiotic insecticides). (4) The substitutions A-to-G at positions 86 and 356 build proteins harboring tyrosine (Tyr) to Cysteine (Cys) mutations in two different regions of BmorCSP4. Base deletion mutations (A< and U<) result in an early stop codon (fsAA*e), thereby yielding shortened proteins. C-to-A mutation changes the position of the stop codon (pmutAA*) and enhances the number of truncated protein isoforms/edited variants [13]. (5) The protein is recomposed not only after the translation process, i.e., when mRNA is translated to produce a protein, but also after protein synthesis. Once the protein is synthesized, the Asparagine-Proline (Asn-Pro) motif switches to another amino acid motif, Aspartate-Arginine (Asp-Arg). The Leucine-Glutamate-Glycine-Lysine (LeuGluGlyLys) motif changes to Phenylalanine-Glutamate-Serine-Glutamate-Lysine-Lysine (PheGluSerGluLysLys) in the C-terminal tail. A Glycine residue (Gly) is inserted next to Cysteine at position 29, 55 or both [13,14,16,17,19,20]. Protein structures are generated by BmorCSP4 templates in SWISS-MODEL using the X-ray crystal structure of MbraCSPA6 (1kx9.1.A) as a reference model [7,18].

Complexity and multi-function in CSPs are largely brought by recent findings about DNA and RNA- polymerization, i.e., specific genetic events on the DNA/RNA template encoding CSP and structure variations [7,8,9,10,11,16,17]. The genetic code dictates the sequence of amino acids in CSP protein, but multiple variant isoforms can exist for a CSP thanks to various post-transcriptional events from intron splicing on RNA in the nucleus to editing of peptide molecules in the ribosome (Figure 1). For instance, using SWISS-MODEL, a software for homology modeling of protein structures and complexes [18], shows that a gene such as *BmorCSP4* (3 exons-2 introns) can lead to eleven protein subtypes, which can be used as templates to produce even more protein subtype variants following various genetic events such as RNA splicing, removal of intron, exon shuffling, RNA editing, protein recoding and protein re-arrangement (Figure 1). Gene, RNA and protein editing could account for the theoretical problem of CSPs interacting with a million or trillion possible ligands, referring not necessarily to the olfactory receptor combinatorial coding theory, but to the recognition, transport and degradation of an enormous amount of potential toxicants and/or all the lipid metabolites that are necessary to activate nuclear receptors, trigger enzymes in different reactions of a chain and/or regulate gene expression in various cellular physiological systems (Figure 1). All of these complementary mechanisms would enable the CSPs to be malleable, i.e., to have a sequence that can be recoded in order to orientate the protein to a new function. The CSP malleability might be crucial, not only for a metabolic tissue such as the gut or the fat body, which certainly needs to degrade a million different xenobiotic chemicals of all sorts, but also for a multi or toti-potent cell on a way to transform in a multitude of organs and tissues or for the multiple synapses establishing new connections in the central and peripheral nervous systems [19].

Four bases, twenty residues, six types of conversion and only four editing enzymes may not be sufficient to underlie the extremely high number of protein variants described in *CSPs* as in the case of *Dscam*, ion channel and cochlear sensory genes [19,21,22,23,24,25]. Genetic variation via splicing and editing mechanisms in immune, neurobiological or sensory systems is probably needed to cause changes in protein families required for the recognition and transport of dozens, hundreds, thousands or millions of potential ligands [26,27]. In particular, we attempt to provide a comprehensive theoretical framework to explore the question of whether different edited versions of a protein such as CSP can be produced to cope with a wide variety of ligands, such as lipids and fatty acids, as well as drug compounds, insecticides and other xenobiotics. It is a hypothesis that is largely compatible with the existence of *CSPs* in different levels within many various kingdoms that contain organisms with cell walls, i.e., arthropods, bacteria, insects and plants [20,28,29,30].

### 1.2. CSPs and Cell Evolution

The *CSP* gene family is not specific to insects and other arthropod classes [20,28,29,30]. The existence of CSP in microbes cannot be a controversial issue. It is unlikely to see microbial samples contaminated by an arthropod tail, an insect scale or some leaf syrup. They are studied as strains that can cause serious infections in the lungs, blood and brain, so they are reared in very controlled areas in aseptic sterile clinical laboratories. It is therefore very unlikely to see an *Acinetobacter baumannii* RNA sample contaminated by a silkworm clone. Bacterial CSP (B-CSP)-RNA sequences have been found not only in Moraxellaceae *A. baumannii*, but also in Enterobacteriaceae *Escherichia coli*, Staphylococcaceae *Macrococcus caseolyticus*, Streptomycetaceae *Kitasatospora griseola*, *K. purpeofusca*, *K. sp. MBT66* and *K. sp. CB01950* and Xanthomonadaceae *Lysobacter capsici* (WP_043907137, WP_1212566, WP_071222707, WP_073810176/WP_083646628, WP_078880044, WP_082558797, WP_089438515, WP_096417339, WP_120787151, WP_120787152, WP_120787167 and WP_120787175) [29,30,31]. Very surprisingly, the very same proteins (BmorCSP2 and BmorCSP6) were found in *Bombyx* and in multi-species in bacteria. BmorCSPs were found not only in bacterial germs of the genus *Acinetobacter*, but also in *E. coli* [20,30,31]. This is an intriguing discovery to discuss function and evolution in the *CSP* gene family. 

CSPs are highly conserved proteins, particularly in the Order Lepidoptera [6,12]. The presence of a same CSP sequence in some bacteria and in a few insect species such as the moths is enigmatic. It is very difficult to conceive that the very same sequence has been conserved for billions of years only in the clades bearing to a few insect genera. Such conserved proteins across two major divisions of life (bacteria and insects) may support the idea of a single universal common ancestor from which every life on earth or every new cell emerged. CSPs seem to be an intriguing coding part in the “dark matter” of the genomes of insects, worms, bacteria and yeasts. These genomes contain many highly conserved sequences whose functions are not yet known [32]. *CSPs* may be essential for the cell’s organization, activity and adaptation, like many other gene families, including transfer RNAs and genes encoding the nucleotide-binding domain of ABC transporters [33]. If an identical protein sequence can be conserved along the evolution from bacteria to insects, it means that more CSP molecules or CSP-like proteins are to be found in the rest of the animal kingdom or that many various organisms have lost *CSP* at crucial steps during their evolutionary history. If the original protein encoded by *BmorCSP2* or *BmorCSP6* gene did not change by an iota despite horizontal gene transfer and the evolutionary change of cell and species over time, it might imply a key function in a basic common universal mechanism of eukaryote and prokaryote cells and in their interactions with an environment that continuously changes. 

The presence of *CSPs* in plants so far appears to be a rather controversial point [34], particularly because plant samples can be easily contaminated by insect eggs, insect scales, insect feathers, many arthropods, fungi and/or bacteria. No efforts have been made to prove the existence of *CSP* in the plant genome. So far, only a rough analysis of plant EST database has been done, urging performing most accurate molecular biology work (molecular cloning of genomic DNA) in order to attest the occurrence of *CSPs* in plants as a fact [34]. This is essential to test the regulation of *CSP* expression in plant species of immense value as source of food or medicine under insecticide-contaminated soil. It could be that plants acquired *CSP* gene by horizontal transfer, but probably not from the insects. Most likely, horizontal gene transfer of *CSPs* occurred not only between microbes and insects or other arthropods, but also between diverse endosymbiotic microbial cells and a variety of plants [20,28,29,30,31,32,33,34].

### 1.3. Genome-Wide Identification, Comparative Genomics and Evolution of CSPs in Hymenoptera

The problem emerges that while insect CSP proteins are stated as being tuned to a high number of diverse functional ligands in many different insect physiological systems [7,8,15,35,36], the *CSP* gene family varies considerably in size across insect species such as moths (herbivorous lepidopteron) and body lice (hematophagous, phthirapteron). Moths retain twenty *CSPs*, while lice display only six *CSP* genes [14,31]. Similar to *Pediculus humanus corporis*, *Drosophila melanogaster* (carpophagous, dipteron) and *Anopheles gambiae* (hematophagous and nectariphagous, dipteron) have a rather low number of *CSPs* (the number of *CSPs (nb)* = 4–to–7 in these species) [12,31,37]. A significantly higher number of *CSP* genes are found in the genome of the red flour beetle, *Tribolium castaneum* (granivorous omnivorous, coleopteron) (*nb* = 19) [14,38]. This includes only model insect species where the genomic organization (clustering, grouping and mapping) of *CSPs* on the chromosomal level is known [12,13,14,31,37,38]. 

**Figure 2:**
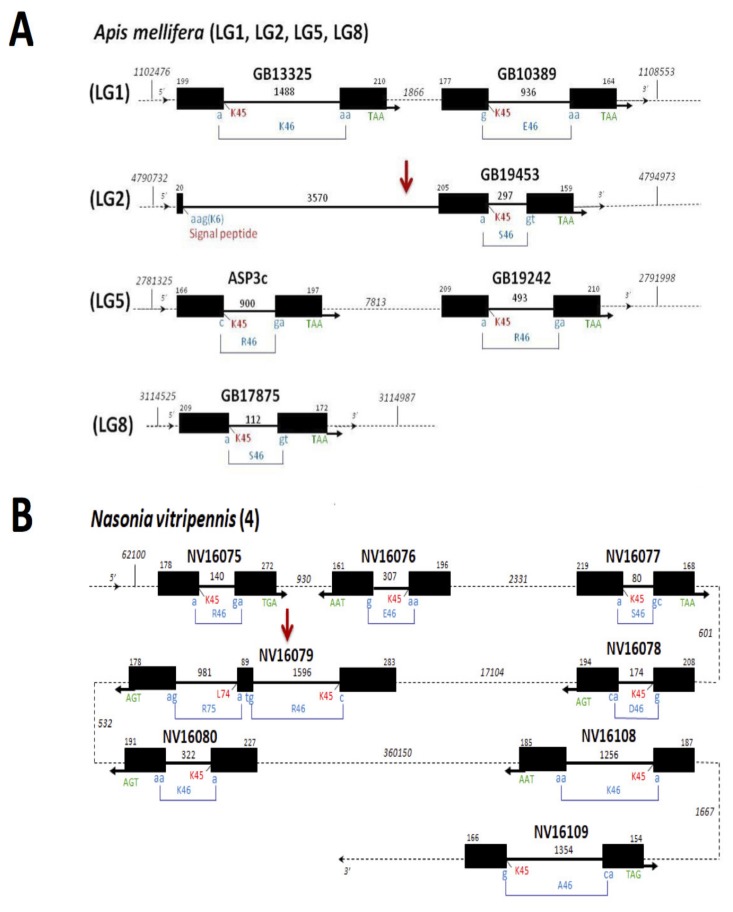
Genomic organization of *Apis* and *Nasonia CSPs*. (**A**) Genomic organization of *A. mellifera CSPs* on four different chromosomes (LG1, LG2, LG5 and LG8) (**B**) Genomic organization of *N. vitripennis CSPs* on chromosome 4. Exons are shown as black boxes, introns as bold black plain lines and intergenic intron regions as dotted lines. The numbers above the box and the plain line give the exon and intron size, respectively. The numbers in italics above the line give the distances between genes. Exon/intron sizes and intergenic distances are given in base pairs. The amino acid residue in red indicates the intron insertion site (K45, L74 or signal peptide). In blue is shown the triplet codon for the amino acid interrupted by intron insertion. Stop codons are indicated in green (*Apis*: TAA; *Nasonia*: TAA or TGA, A>G switch in stop codon). Horizontal arrow in black indicates the orientation of the gene: 5’–3’ (right) or 3’–5’ (left). The red vertical arrow points out the different position of the *double-intron CSP* gene in *A. mellifera* and *N. vitripennis*, respectively.

This poses the question of whether adapting and developing new phenotypes, the number of *CSPs* in insects and/or other arthropod species depends on the feeding habits and host preferences. Here, adding new sets of data in comparative genomics of a handful of species limited to two dipterons, one lepidopteron, one coleopteron and one phthirapteron were required to address hypothesis analysis. Therefore, we selected two cases in eusocial insect species (bees and wasps) to add two hymenopterons in the handful of species for which genome organization and structure of *CSPs* in insects are known [12,13,14,31,37,38]. We characterize hymenopteron’s *CSPs*, annotation, classification, genomic organization, structure, phylogenetic distribution and expression of the *CSP* genes from honeybees (*Apis mellifera*) and parasitoid emerald jewel wasps (*Nasonia vitripennis*). In particular, we show that both *A. mellifera* and *N. vitripennis* have an extremely low number of *CSP* genes, as found, for instance, in the *Drosophila* fly, the *Anopheles* mosquito and the *Pediculus humanus corporis* louse (see Figure 2, Tables S1,S2) [12,13,14,31,37,38].

Although both species used as models, *A. mellifera* and *N. vitripennis*, do belong to the order Hymenoptera suborder Apocrita, they are part of two different clades, i.e., Aculeata and Parasitica that diverged more than 200 Mya. Key differences are found in their physical and behavioral characteristics. Parasitoid wasps such as *N. vitripennis* parasitize Diptera, mainly on the families Calliphoridae and Sarcophagidae. They seek out prey, kill the pupae they attack and lay eggs in the target host fly. So, the search of parasitic wasps is for oviposition. This behavior makes the wasps very distant from the honeybees that seek nectar and pollen from flowers and flowering plants. It is therefore of particular interest to compare their genomic variations in regards to *CSPs* to check whether *CSP* could be a mechanism by which behavioral or physiological characteristics of *Apis*, *Nasonia* and other model insect species have changed. 

In the initial analysis of the honeybee genome, the existence of six *CSPs* has been reported in *A. mellifera*: six *single-intron* structures [37,39,40]. Analyzing a new assembly (sequence update) of the honeybee genome [41], we confirm the existence of only six *CSPs* in bees (*AmelASP3c*, *AmelGB10389*, *AmelGB13325*, *AmelGB17875*, *AmelGB19242* and *AmelGB19453*) and localize them on specific chromosomes (Figure 2A, Table S1). However, we find that there is an additional intron inserted in the signal peptide of *GB19453*. Bee *CSPs* are five *single-intron* structures and one *3 exons-two introns* structure (Figure 2A), not six *single-intron* structures as reported in the initial analysis [37,39,40]. In contrast to *A. mellifera CSPs*, no genomic data have ever been reported about *N. vitripennis CSPs*. Ten sequences encoding CSPs have been reported from the analysis of a cDNA library from the jewel wasp [42]. Here, we show that the number of ESTs encoding CSPs do not reflect the number of *CSPs*, as expected for a protein gene family with RNA variance like *CSP* genes. Here, we have performed a cautious genome analysis to precisely assess the number of genes encoding CSPs in the parasitoid jewel wasp [43]. Analyzing the assembly (sequence update) of the parasitoid wasp genome [43], we find only eight genes encoding CSPs: *NV46080*, *NV16108*, *NV16109*, *NV16075*, *NV16076*, *NV16077*, *NV16078* and *NV16079* (Figure 2B, Table S2). Therefore, after a cautious comparative analysis of the new assembly of *Apis* and *Nasonia* genomes, we report that these two hymenopteran species retain only six and eight *CSP* genes, respectively. 

Interestingly, in contrast to their counterparts in *Tribolium* and *Bombyx*, *Apis* and *Nasonia CSPs* are all functional genes. In *Apis* and *Nasonia*, there are no pseudos or truncated *CSP* genes that have lost function after exon deletion [14,38] (Figure 2, Figure S1 and Tables S1,S2). Moreover, in contrast to coleopteran, dipteran and lepidopteran species, no intronless *CSP* genes are found in the honeybee *A. mellifera* and the parasitoid jewel wasp *N. vitripennis*, similar to the human body louse *P. humanus* [12,13,14,30,37,38] (Figure 2 and Tables S1,S2). Therefore, the data presented here argue for *CSP* loss as an essential evolutionary mechanism for adaptation and phenotypic variance not only in lice, but also in bees and parasitoid wasps. 

Comparing *CSP* gene structures between *Apis* and *Nasonia*, we find that the number of *3 exons-2 introns (3e2i)* genes is the same (= 1), but the number of *single intron* genes is superior in *Nasonia* (+2; Figure 2 and Figure S1 and Tables S1 and S2). Silk moths have three *3e2i* genes among twenty *CSPs* [14]. The repertoire of *CSPs* in fruit flies is limited to two intronless and two small single intron genes. No *3e2i* gene structures are found among the four *CSPs from D. melanogaster* [12,37]. From the analysis published in Wanner et al., there are apparently also no *3e2i CSP* genes in the mosquito *A. gambiae* [37]. So, evidence in genomic analysis suggests that the number of *3e2i CSP* genes varies across insect species. Interestingly, beetles and lice are also known to retain only one *3e2i CSP* gene [30,38]. However, while beetles accumulated *CSPs* [38], the same arrangement of *CSPs* is maintained in lice and bees. *Pediculus CSPs* are one *3e2i* gene and five small *single intron* genes [30], as found for the honeybee (Figure 2A). Importantly, *Apis* strongly differs from *Nasonia* that shows a completely different genomic organization in *CSP* genes (see Figure 2). In *Apis*, they are clearly divided into four groups that are located on four different chromosomes (Figure 2A), while the *CSPs* are organized in the same small cluster of genes on chromosome 4 in *N. vitripennis* (Figure 2B). *GB13325/GB10389*, *GB19453*, *ASP3c/GB19242* and *GB17875* are located on chromosome LG1, LG2, LG5 and LG8, respectively (Figure 2A and Table S1). *GB13325* and *GB10389* are found near each other on LG1, while *ASP3c* and *GB19242* are found near each other on LG5. Furthermore, in bees, all *CSP* genes or pairs of *CSP* genes are found with the same transcriptional direction (5’–3’). They are about the same size (about 1 Kb) and differ only in intron size (from 112 to 3570 bps). Intron is always located at the same position, after Lys45, after the first base of the codon for amino acid at position 46 (Figure 2A). They all have TAA stop codon. Therefore, they might represent successive genome duplications, as described for the red flour beetle *T. castaneum* genome (beetlebase) [38] (see Figure 2A and Figure S1). Apparently, the duplicated copies of *GB19453* and *GB17875* were lost following genome duplication [41]; they are found as single genes on LG2 and LG8, respectively (Figure 2A). 

On the contrary, *CSPs* occur in pairs and the members of each *CSP* pair are TGA and TAA-stop codon in the parasitoid jewel wasp *N. vitripennis*. Paired *CSP* genes are found in the opposite direction. The genes in the second group of *Nasonia CSPs* are oriented in an opposite direction (3’–5’) compared to *NV16079* (Figure 2B). Therefore, the *CSPs* from jewel wasps might originate from inverted gene duplication, which is in strong contrast with the *CSPs* from the bees. 

This may be correlated with the position of *double intron* genes within the *CSP* gene cluster in *Apis* and *Nasonia*, respectively. In *Apis*, *GB19453* is located distantly from the other *CSPs* on a separated chromosome, while *NV16079* is located right in the middle of the *CSP* gene cluster on the same chromosome (chromosome 4) in *Nasonia* (Figure 2, red arrow). In the body of louse *P. humanus corporis*, the *double intron CSP* gene (*Phum594410*) is located farther away from the other *CSP* genes, as also found in *T. castaneum* [31,38]. This can also be found in *B. mori* where the three *3e2i CSPs* are located very distantly from each other [14]. Therefore, it is very unlikely that *3e2i CSP* genes come from the same common duplication event after analyzing the current handful of species (*Anopheles*, *Bombyx*, *Drosophila*, *Pediculus* and *Tribolium*) compared to our new data in *Apis* and *Nasonia.*

Furthermore, *Bombyx CSPs* are either TAG or TAA stop codon [13,14]. *Tribolium CSPs* are all TAA-stop codon [38]. *Pediculus CSP* genes are either TGA or TAA-stop codon, but not in pairs [31]. All these differences among stop codons, gene structures and genomic/chromosomal distributions show that *CSPs* from flour beetles, flies, moths, mosquitoes, lice and Hymenopteran species such as honeybees and jewel wasps have been subjected to different evolutionary paths that led to very specific genetic repertoires. This may reflect a unique evolutionary history for each insect lineage and suggest how the biology, the shape and the behavior exert strong influences on the evolution of the *CSP* repertoire. 

Intron insertions occurred after the first base of the codon for amino acid 46, except for insertion in signal peptide (Figure 2). Interestingly, we find that in *CSP* genes such as *GB19453*, one intron is inserted only a few nucleotides after the start codon encoding the amino acid methionine (Figure 2A). The same observation (intron1 inserted shortly after the start of the signal peptide) was made in *AAJJ1196A* and *BmorCSP19* (Figure S1) [14,38,41,42,43]. In the case of these genes, the intron is inserted after the third base and therefore does not cause codon disruption (phase 0 intron). Phase 0 intron1 position suggests that splicing of the signal peptide region is tightly regulated and that the length of the signal peptide is functionally important in *CSPs*. 

In addition, the intron is always inserted squarely in the middle of the *CSP* gene, between the two nucleotides that make up codon positions 1 and 2 in a specific codon that codes for amino acid 46 [14,30,37,38]. This is also observed in *CSPs* from honeybees and parasitoid wasps (Figure 2). In both species, the intron from *CSP* is located after the first base and disrupts the codon (phase 1 intron). Amino acid 46 can be Glu, Lys, Arg and Ser in the honeybee (Figure 2A). It can be Arg, Glu, Ser, Asp, Lys and Ala in the parasitoid wasp (Figure 2B). Therefore, it seems to be a widespread general view that the intron in a *CSP* gene contributes to the variability in amino acid 46 and requires very specific splicing mechanisms to avoid cutting a functional domain in CSP protein. Apparently, the intron boundaries of *CSPs* in many insect species indicate that the codon for amino acid 46 is a crucial site to underlie evolution and protein diversity in the *CSP* family. 

Furthermore, we find that the insect genome seems to provide a simple form of sequence recovery (or backup). We find that the amino acid 46 is also coded by the three nucleotides at the tip of intron1 and intron2. All nucleotide combinations that code for amino acid 46 are found at the intron insertion site. Thus, *CSP* genes could be spliced at different codon positions without altering the primary amino acid composition of the CSP protein in any way. This is the case of *NV16077* where Ser46 can be encoded not only by *AGC* (disrupted codon), but also by *AGT* found at the intron1 boundary. This is also the case for *NV16079* where Arg75 can be encoded not only by *AGA* (disrupted codon), but also by *AGG* found in intron2 boundary. Therefore, a very important role is played by the codons at the intron boundary of *CSPs* to allow protein diversity.

Curiously, in our analysis, we find that there are no large introns containing a copy of gene or a retroposon in bee and wasp *CSPs*, in contrast to beetles and moths [14,38] (Figure 2, Table S1). The same situation has been described in human body lice [31]. *Pediculus CSP* genes (*PhumCSPs*) are all characterized by very short intron lengths (<288 bps) and all lack retroposon [31]. We find that the honeybee *A. mellifera* and the jewel wasp *N. vitripennis CSP* genes have introns varying in size between 80 and 3570 bps. The largest intron is intron2 from *GB19453* (Figure 2, Table S1). Importantly, *GB19453* and *NV16079* genes differ much, not only in intron size, but also in the position of intron boundaries. Introns in *GB19453* inserted after signal peptide and Lysine at position 45 (K45), respectively, while in *NV16079* they inserted after K45 and Arginine at position 75, respectively (Figure 2). This shows that despite a common exon–intron structure, these two genes do not originate from the duplication of a common ancestor, but rather from intron insertions that occurred independently in Apidae (honeybees) and Pteromalidae (parasitoids) during the course of evolution in the order Hymenoptera. Intron insertion also occurred independently in Lepidoptera as *BmorCSP10*, *BmorCSP14* and *BmorCSP19* show distinct intron boundaries (Figure S1) [14]. However, some specific *CSP* genes such as *AmelGB19453*, *AAJJ1796A*, *BmorCSP19* and *Phum594410* show the very same intron boundaries (intron1 inserted in signal peptide and intron 2 inserted after Lys45), strongly suggesting that insertion of intron1 in the signal peptide of *CSPs* occurred before the split of Hymenoptera, Coleoptera, Lepidoptera and Phthiraptera (parasites) [14,31,38] (see Figure 2A). There are no such *double-intron CSPs* in a parasitoid chalcid insect species such as the emerald jewel wasp *N. vitripennis* (see Figure 2B), which may indicate that this ancestral *double-intron CSP* gene was present in the last common ancestor of bees, beetles, moths and lice (i.e., more than 400 Mya), but was lost later during evolution in particular in parasitoids and other groups of predatory insects. 

We reveal a high level of genetic plasticity in *CSPs*, which would be essential for evolutionary adaptation. This gene family is characterized by introns of different phases that inserted at different periods during the course of evolution in the insects. Some introns inserted at an early stage of evolution and were conserved even after the separation of the different insect lineages. Second intron inserted at a later stage of evolution, but was lost in some specific lineages, including the parasitoid lineage. We also reveal that gene duplication profiling within the *CSP* group is very different between honeybees (characterized by chromosomal duplication) and parasitoid wasps (characterized by extensive local inverted duplication), suggesting that the evolution of *CSP* genes may contribute to the development of very specific phenotypes and/or behavioral traits not only in hymenoptera, but also across many various organisms from bacteria to hymenoptera.

## 2. Phylogenetic Distribution Analysis in Insects and Bacteria

**Figure 3:**
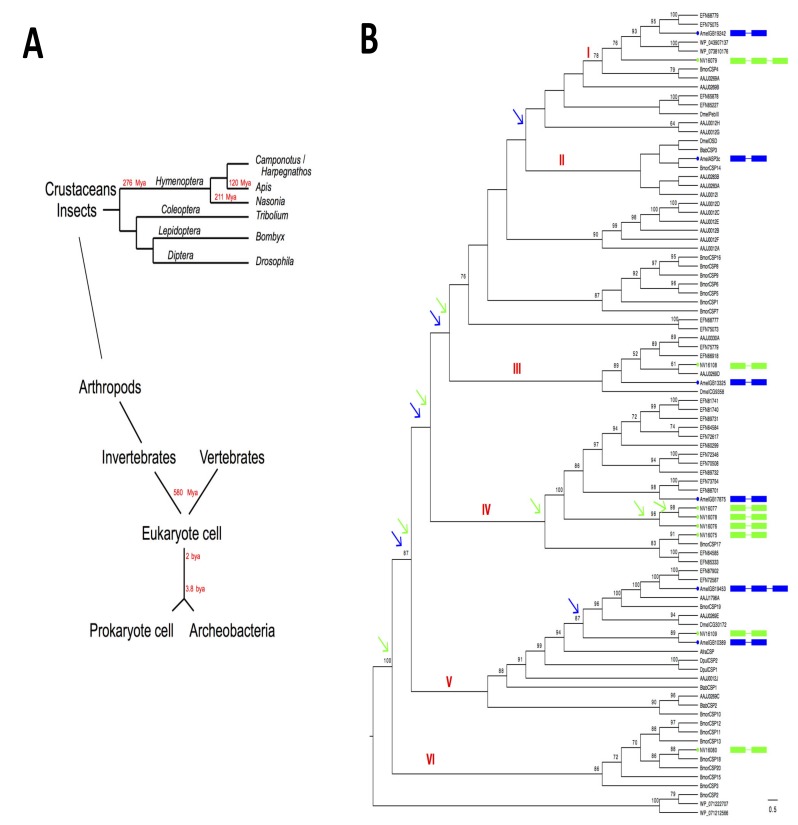
Schema of relationships from bacteria to insects and amino acid phylogenetic analysis of *Apis* and *Nasonia CSPs*. (**A**) Timeline of the evolutionary history of life from bacteria and prokaryote cells to multiple species of insects. (**B**) Gene phylogeny and orthology groups of bacterial/insect *CSPs* with focus on gene duplication profiling in honeybees and jewel wasps. Bacteria: *Acinetobacter* (WP_071212566, WP_071222707); *Kitasatospora* (WP_04307137, WP_07383810176) [29,30]. Insects: ants (EFN), beetles (AAJJ), flies (Dmel), moths (Bmor) and whiteflies (Btab) [12,13,14,15,37,38,44,45,46]. Crustacean: *A. franciscana* (AfraCSP; ABY62736, ABY62738); *D. pulex* (DpulCSP1, DpulCSP2; ABH88167, ABH88166). Phylogenetic trees are generated from a total of ninety protein sequences (IQ-TREE, UFBoot; 1000 replicates). Blue and green color circles represent *Apis mellifera* (*Amel*) and *Nasonia vitripennis* (*NV*) protein sequences, respectively. The gene structures are shown on the right for *Amel* (in blue) and *NV* (in green) *CSPs*. Branches are shown supported by >50% bootstrap value. Six major orthology groups are found corresponding to specific *Amel* and *NV* CSP sequences: group I (AmelGB19242, NV16079); group II (AmelASP3c); group III (AmelGB13325, NV16108); group IV (AmelGB17875, NV16075, NV16076, NV16077, NV16078); group V (AmelGB10389, AmelGB10453, NV16109); group VI (NV16080). Blue and green arrows indicate gene duplication profiling in *Amel* and *NV*, respectively. Supplementary Methods: Figure 3 The multiple sequence alignment was performed using Muscle global alignment (www.ebi.ac.uk/Tools/msa/muscle). Phylogenetic trees were constructed using IQ-TREE (http://iqtree.cibiv.univie.ac.at). The following parameters were used for phylogenetic tree construction, ultrafast bootstrap (UFBoot, using the –bb option of 1000 replicates), and a standard substitution model (-m TEST) was given for tree inference. The generated trees from IQ-TREE tool were visualized using Figtree (http://tree.bio.ed.ac.uk/software/figtree) and the branch-support values were recorded from the output treefile. The re-rooting was performed on WP_071212566 and WP_071222707 node. The trees were modified as cladogram and increasing order nodes were applied under trees section for better visualization.

To measure the proportion of phenotypic variance attributable to genetic variance in *CSPs*, we analyzed the timeline of the evolutionary history of life from bacteria to insects and performed a phylogenetic analysis of the amino acid sequences using bacterial and insect *CSPs*. Our analysis shows that multiple duplications have taken place throughout the history of the gene family and eventually that, some of these duplications are unique to all hymenopteran species such as ants, bees and parasitoid wasps [44,45], while others are more ancient and are shared between various insect and bacterial orders (Figure 3). 

In our phylogenetic analysis of *CSPs* from bacteria to insects, we also used the *CSPs* from *D. melanogaster* and *B. tabaci* as taxa since it was shown that dipteran and homopteran CSPs play a key role in insect defense [15,46]. Whiteflies such as *B. tabaci* show little in common with the pupal development of holometabolous insects (ants, bees, beetles, flies, moths and wasps). *Bemisia* is characterized by incomplete metamorphosis (hemimetabolous insect). The nymph resembles the adult in form and eating habits; there is no pupal stage in *B. tabaci*. The relationship of the bacterial and insect CSPs was studied with maximum parsimony (MP) analysis; MP was used to establish strict consensus trees using the IQ-TREE algorithm as described in Xuan et al. [16] (Figure 3).

In agreement with the phylogenetic distances between *Camponotus/Harpegnatos*, *Apis* and *Nasonia* (Figure 3A), our phylogenetic analysis shows that Hymenopteran CSPs such as EFN68779, EFN75075, AmelGB19242 and NV16079 are closely related; they form a group (group I) with a significant bootstrap value (78%; Figure 3B). Group I also includes *Bombyx* CSP4, *Tribolium* AAJJ0269A and two CSPs from the Streptomyces *Kitasatospora* (Figure 3B). The two CSP sequences from *Kitasatospora* bacterial strains (WP_04307137; WP_07383810176) fall close to NV16079 and AmelGB19242, showing a group of CSPs conserved from bacteria to insects. This group is clearly indicative of common ancestry between insect and bacterial *CSPs* [30,31]. However, AmelASP3c is more distantly related to this group I. AmelASP3c helps build another group of CSPs (group II), which also includes DmelOSD, BtabCSP3 (known to bind plant oil), BmorCSP14 and three Coleopteran CSPs, namely AAJJ0283B, AAJJ0283A and AAJJ0012I (Figure 3B). 

The position on the tree (and gene structure) of *AmelGB19242* and *AmelASP3c* suggests that these two genes come from the same gene duplication that has happened before the split of Hymenoptera, Lepidoptera and Coleoptera, i.e., more than 300 Mya. Interestingly, there are no *Camponotus*, *Harpegnatos* or *Nasonia* clades in group II (Figure 3B), suggesting that this duplication event happened before the divergence of hymenopteran species, or that *AmelASP3c* gene has been lost in hymenopteran species such as the wood carpenter ants (*C. floridanus*), the predator jumping ants (*H. saltator*) or the parasitoid jewel wasps (*N. vitripennis*). 

The wasp gene *NV16108* is clearly orthologous to Coleopteran *AAJJ0269D* gene (61% bootstrap). The two genes fall in a third group (group III) together with *AAJJ0330A*, *EFN75779*, *EFN66918*, *DmelCG9358* and *AmelGB13325* (89% bootstrap; Figure 3B). So, *NV16108* and *AmelGB13325* might originate from the same old gene duplication that took place in the far common ancestor of honeybees (Aculeata) and parasitoid wasps (Parasitica). In contrast, *NV16075*, *NV16076*, *NV16077* and *NV16078* might be the result of a series of much more recent gene duplications that specifically happened in Parasitica (group IV). These four genes seem to have been essential for the birth and evolution of the tiny parasitoid wasp, *N. vitripennis.* They labelled split-specific branches in *NV* with significantly high bootstrap values (96–98%; see green arrows, Figure 3B). 

A larger group of *CSP* orthologs (group V) groups the honeybee genes *AmelGB19453* and *AmelGB10389* together with wasp *NV16109*, ant *EFN87902*/*EFN72587*, *Tribolium AAJJ1796A*, *AAJJ0269A* and *AAJJ0269E*, as well as the amino acid sequences for *Bombyx BmorCSP19* and *Drosophila DmelCG30172*. This group does not only include *CSP* genes from holometabolous insects, but includes also some genes expressed during the embryonic development in crustaceans (*AfraCSP*, *DpulCSP1* and *DpulCSP2*) and *B. tabaci chemosensory protein type 1* (*BtabCSP1*). BtabCSP1 is known to transport lipids such as linoleic acid (LA or C18:2 fatty acid) [15], suggesting that the main function of these CSPs from group V is to transport long fatty acid lipid chains such as C18:2. Using MP analysis, high bootstrap values (close to 100%) mean uniform support with BtabCSP1. All the characters informative enough to define group V agree that BtabCSP1 and other CSPs in this group are related with a common biological function (Figure 3B). 

While *NV16109* and *AmelGB10389* are clearly two orthologous copies of the same gene (89% bootstrap value), *AmelGB19453* has no orthologous copy in *N. vitripennis*, begging the question of whether this absence is due to recent gene loss in some specific clades of the order Hymenoptera, similar to *AmelASP3c* (Figure 3B). *NV16080* forms an orthology group including several *BmorCSPs* but neither bee *CSP* family genes nor beetle *CSPs* are found in this group (group VI; Figure 3B). *BtabCSP1* and *BtabCSP2* (related to cinnamaldehyde transport) arose from a gene duplication that occurred in whiteflies, beetles and moths, but not in hymenopteran species [15,28] (Figure 3B). Therefore, the phenotype associated with plant-feeding habits and resistance to plant toxins seems to be associated with genetic variation, genetic changes, gene rearrangement and/or plasticity in some very specific groups of *CSPs*. 

This poses the question of whether *CSPs* have contributed to the development of the eukaryote cell, the insect cell, as well as the bacterial prokaryote cell. The eukaryote cell divided into invertebrates and vertebrates about 580 Mya. The eukaryote cell was built about 2 Bya(Billion years ago), and the original archeobacterium and/or the prokaryote cell evolved 3.8 Bya (Figure 3A). The six orthology groups revealed in our phylogenetic analysis of bacterial/insect CSPs always display counterparts from various insect orders such as Coleoptera, Lepidoptera and/or Diptera, and in some cases, they also display a number of clades from the bacteria superkingdom. This indicates that *CSPs* originate from an extremely ancient duplication, which probably occurred prior to the origin of insects, much before the different insect orders took place (e.g., about >350–412 Mya). The first *CSP* gene duplication probably took place in some archeobacteria some billion years ago, perhaps approximately when life and diversity had to come from the original cell. So, the evolution and editing process in *CSPs* could date back to Bya and may eventually help develop an understanding of, not only cell fate and/or organismal evolution in various prokaryote systems, but also neural development and/or birth and evolution in highly diverse groups of eukaryote animal species. Interestingly, while it contains *AmelGB17875*, *BmorCSP17* and multiple copies of *Camponotus*, *Harpegnatos* and *Nasonia CSPs*, group IV lacks *Drosophila* and *Tribolium* clades, strongly suggesting that this gene has been subjected to continuous series of duplications in ants and parasitoid wasps, but has been lost specifically in Diptera and Coleoptera. Some *CSP* genes are more recent than others; some represent duplicates that occur specifically in Apocrita, but none of the six orthology groups that we describe here happen to be specific to bees, wasps or Hymenoptera (Groups I-VI; Figure 3B).

In addition, in our study, we find that the *3e2i*/*double-introns CSP* genes from *A. mellifera* and *N. vitripennis* (*AmelGB19453* and *NV16079*) group separately, confirming our first assumption that duplications as well as intron insertions have occurred independently in Aculeata (*Amel*) and Parasitica (*NV*), respectively ( Figure 2; Figure 3). However, our most intriguing finding might be that bacterial *CSP* sequences such as *WP_071212566* and *WP_071222707* from Acinetobacter *A. baumannii* fall at the bottom of the phylogenetic tree, together with *NV16080* and multiple *CSP* sequences from the silkworm *B. mori* (*BmorCSP3*, *BmorCSP11*, *BmorCSP12*, *BmorCSP13*, *BmorCSP15*, *BmorCSP18* and *BmorCSP20*; Group VI: 86% bootstrap value). Importantly, we note that *B. mori* CSP2 sequence is identical to bacterial “CSPs” WP_071212566 and WP_071222707 (100% bootstrap), strongly suggesting that this set of proteins represents the most ancient form in the CSP family and an extremely old molecule, as well as being perhaps the most ancient type of carrier molecule in the earliest known life forms on Earth (back to >3 Bya; Figure 3AB). *CSP* gene duplicates can evolve so as to parse the original function or to acquire new roles. In our phylogenetic analysis, *NV16080* is orthologous to *BmorCSP18*, which is a truncated gene in the silkworm moth *B. mori* [14] (Figure 3B). So, it seems that the original function of this gene has been lost in silkworm, but multiple derived duplicated versions of *CSP18* have taken on the role of specifying moth identity. In contrast, loss of *NV16080* rather seems to have been decisive for the development of many other insect lineages such as ants, bees, beetles and flies (Figure 3). 

**Figure 4:**
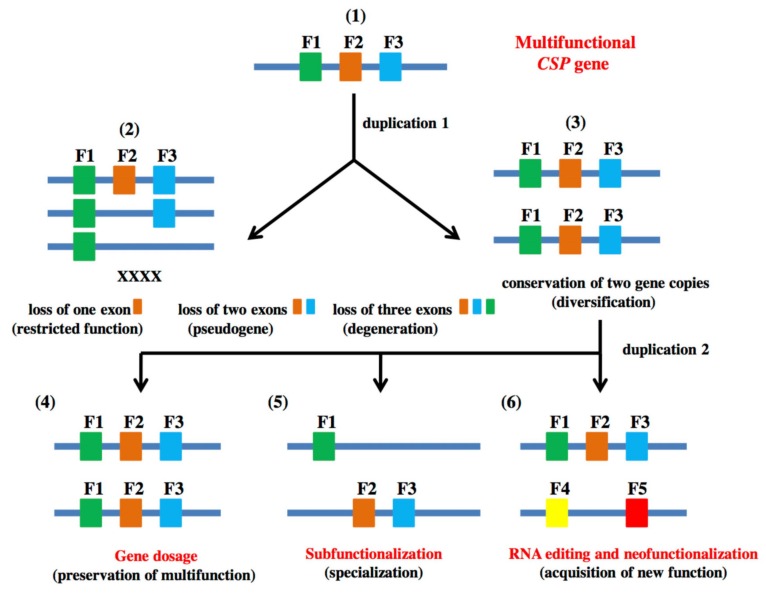
Evolution of *CSPs* for neofunctionalization. (**1**) At some point far back in time (Bya), the original ancestor *CSP* gene retains three functions (three exons: F1, F2 and F3). The outcome of the first early duplication (duplication 1) is a pair of tandem paralogous genes strictly identical to the original gene. (**2**) Duplication 1 can lead to loss of one exon (restricted function), loss of two exons (unexpressed pseudogene: *BmorCSP5*, *BmorCSP16*, *BmorCSP18* and *AAJJ0269A1B* [14,38]), or loss of the three exons (degeneration/loss of a complete gene copy). (**3**) Duplication 1 can also lead to the conservation of the two gene copies, increasing the original functions while providing a template for CSP diversification. Further successive genome duplications (duplication 2) allow expansion of the gene family. (**4**) Additional copies of the *CSP* gene are preserved and keep performing the same functions (F1–F3) as the ancestral gene, thus amplifying the original activity of “*CSP*”. (**5**) The different functions of *CSP* are divided over specific duplicated copies, leading to CSPs with more specialized functions (subfunctionalization). (6) Other duplicates are subjected to RNA editing events and multiple specific mutations that lead the *CSP* family to acquire new genes and functions (F4, F5).

Therefore, in our study, comparative genomics and phylogenetic analysis both show that *CSP* genes evolved through duplication and that many duplicated *CSP* genes had different fates as found not only in beetles, lice and moths, but also in honeybees and parasitoid wasps [14,31,38] (see Figure 2 and Figure 3). The most common outcome of duplication in *CSPs* is loss of the duplicated copy as we found in our analysis of hymenopteran *CSPs* (Figure 2 and Figure 3). Then, there can be three different scenarios if the two duplicated copies are conserved following the gene dosage phenomenon described in the model eukaryote *Saccharomyces cerevisiae* [47] (Figure 4). In gene dosage, the two gene copies keep performing the same function as the ancestral gene and thereby introduce increased activity of the gene. Here it is a gene dosage phenomenon, i.e., the need for duplication events for sharing functions. However, duplication can also lead to restricted function or complete loss of function (Figure 4). The two events seem to have happened in the *CSP* family. Most of *CSPs* are *single-intron* genes, thus representing more restricted function [14,15,27,30,38] (also see Figure 2 and Figure 3). Other *CSPs* are truncated unexpressed pseudogenes as found in *B. mori* and *T. castaneum* [15,38]. At a later stage of evolution, the different functions can be divided over some additional successive duplications (subfunctionalization or functional specialization of the two gene copies). Then, with one duplicated copy still performing the original function of the ancestor gene, some other new copies of the gene were subjected to mutations through or mediated via RNA editing and acquired new functions as described in Lepidoptera [13,16,17,19,20]. In particular, some specific RNA variant isoforms may have returned to the genome through or via retrotransposition to drive evolution in some groups of genes as proposed for *Bombyx CSPs* [16,20] (Figure 4).

## 3. *CSP* Gene Expression in Response to Environmental Change

Not only the knowledge of how CSP-encoding genes have evolved, but also their tissue expression profiling are important to solve the function of the protein. For instance, CSP expression is detected during early embryonic development stages of the brine shrimp *Artemia franciscana*, clearly rejecting a function in olfaction for this protein [48]. The brine shrimp is a micro-crustacean rather known for producing cysts (dormant eggs) well adapted to harsh and critical life conditions. The CSP protein family is commonly found in many various organisms from bacteria to insects and crustaceans, including marine arthropods (that do not respond to airborne odor volatiles), and certainly they have a crucial role to play in the molecular mechanisms underlying adaptation to new environments, rather than olfaction. 

Organismal adaptation to a new environment may start with very general metabolic pathways leading, for instance, to the degradation of toxic xenobiotic factors. Bacteria have no neurons and no olfactory receptors, but they are capable of chemotaxis, i.e., they can redirect their movements in the presence of chemical (amino acid or sugar) gradients [49]. Multiple *CSPs* are expressed in many various bacterial strains such as *A. baumannii*, *K. griseola*, *K. purpeofusca*, *K. CB01950*, *K. MBT66*, *E. coli* and *M. caseolyticus* [30] (Figure 3), but their role in binding solute ligands such as amino acids or sugars as well as their obvious presence in the “olfactory” hedonics of bacteria are far to be proved. Meanwhile, most bacterial species are known to readily adapt to their new environments and to develop multiple ways of multidrug chemical resistance. Therefore, studying *CSPs* may significantly help test the hypothesis that this family of genes is particularly crucial for adaptation mechanisms and evolution of cells. The accumulation of data in insect *CSPs*, in particular in moths, can now help us provide a remarkable insight into the hypothesis that the genetic plasticity in *CSPs* underlies cell fate and evolution.

The insect EST database, consisting of more than thirty thousands of mRNA sequences from *n* tissue libraries, by definition, contains information for the association of genes with tissues of origin. EST profiles in the bee *A. mellifera* do not show *CSP* gene expression restricted to the “olfactory” or “chemosensory” system. They show gene expression patterns for *CSPs* in (1) the head, (2) the brain, (3) the antennae, and 4) the whole body [39,40] (Table S3). Similarly, *CSPs* from the tiny wasp *N. vitripennis* are not expressed only at the adult stage, but they also express in larvae, prepupae and pupae [42,43]. More than 200 EST sequences are reported in the whole body of the fly *D. melanogaster* for *pebIII* and *CG9358 CSP* genes [50,51,52]. 

This is consistent with pioneer Northern blot experiments showing that moth *CSPs* are highly expressed not only in the antennae, but also in the legs, as well as in the three main parts of the insect body, head, thorax and abdomen [4,5,6]. The analysis of EST sequence database in the silkworm moth *B. mori* (KAIKObase) shows that the EST-cDNAs encoding BmorCSP are very abundant in antennal tissues, the compound eyes (the ocelli supply insect vision), the midgut, the ovaries, the fat body and the female pheromone gland. In the silkworm larvae, *CSPs* are found to be expressed in many various different types of tissues such as the hemocytes, the testis, the posterior silk gland, the epidermis and the maxillary galea (the sensory mouth part of the larva) [53]. Therefore, the distribution of ESTs encoding CSPs shows that these proteins are broadly expressed in early and late stages of the developing insect and absolutely never maintain a specific domain of sensory, non-sensory or neural tissues, in particular in adults.

**Figure 5:**
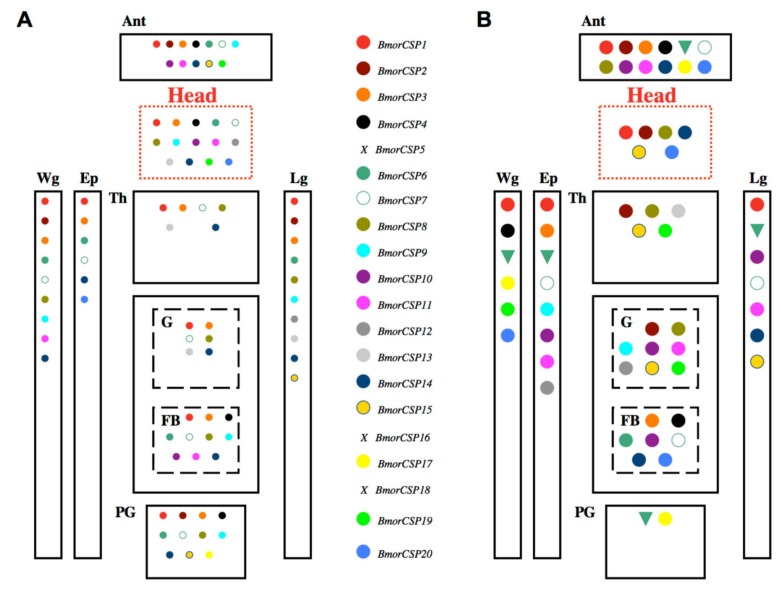
Tissue expression profiling of *CSPs*. *B. mori CSP* gene expression profiling under normal conditions (**A**) and following exposure to abamectin insecticide (**B**). Data are from Xuan et al. [13,14]. Specific gene expression is shown by color code. X indicates no expression for truncated genes (*BmorCSP5*, *BmorCSP16* and *BmorCSP18*). Up regulation in the expression levels of *CSP* genes is indicated by a larger circle. Down regulation in the expression levels of *CSP* gene (*BmorCSP6*) is indicated by a triangle oriented down. Ant: Antennae, Ep: Epidermis, FB: Fat Body, G: Gut, Lg: Legs, PG: Pheromone Gland, Th: Thorax, Wg: Wings.

Importantly, a more detailed gene expression study focusing on each gene in the *BmorCSP* family using real-time PCR showed unequivocally that all *CSPs* are expressed in all various tissues at the adult stage [14]. This is in agreement with the finding of wide expression of *BtabCSP1* across many different adult tissues in the whitefly *B. tabaci* and the binding of the protein to a fatty acid molecule such as C18:2 lipid, linoleic acid [15]. The molecular study from Xuan et al. comparing gene expression of all of the twenty *CSPs* from the silkworm moth *B. mori* (seventeen functional genes, three truncated pseudo-genes: *BmorCSP5*, *BmorCSP16*, *BmorCSP18*) is very important in an analysis of the role of *CSPs* in cell adaptation for three reasons: the results show that (1) none of the *BmorCSPs* is specifically expressed in one given tissue, (2) all the *BmorCSPs* are widely distributed across the insect body and (3) about all of the seventeen functional *BmorCSP* genes show higher expression following exposure to abamectin insecticides. This strongly suggests a function in relation with immune responses, in particular in xenobiotics degradation for the whole *CSP* family (Figure 5). The three truncated genes (*BmorCSP5*, *BmorCSP16*, *BmorCSP18*) are not expressed in all tissues investigated (antennae, legs, head, pheromone gland, wings, thorax, epidermis, fat body and gut), demonstrating the loss of function after truncation of a duplicated gene [14].

Most *CSPs* are expressed under control conditions in a tissue-specific manner, none of the genes is consistently restricted to a common single tissue, and the expression of the whole group of *BmorCSPs* is drastically increased in a tissue-specific manner in response to a chemical stress such as the exposure to an insecticide molecule (Figure 5). It seems like a metabolic chain that enrolls most *CSPs* in the same process, i.e., the same fueling system that is essential for many various cells, organs and tissues from an organism even under normal conditions, i.e., no change of environment. Our previous study in moths shows that under no chemical or viral stress conditions, twelve to fourteen *CSP* genes are expressed in the head and peripheral organs, but their expression is never restricted to nerves or sensory tissues. About six to nine *CSP* genes are mainly expressed in the epidermis, thorax and/or the gut tract, definitely rejecting a function tuned to olfaction, chemosensing or chemotaxis. About ten to eleven *CSP* genes are mainly expressed in the pheromone gland and fat body, which are two crucial organs for mechanisms involved in lipid fatty acid uptake, metabolism, transport and trafficking (Figure 5) [14]. Free lipids and fatty acids are essential as fuel molecules for cells to regulate activities such as hormone biosynthesis, digestion, locomotion, flying, pheromone production, insecticide xenobiotic degradation and/or various immunological responses to bacterial/viral infection or host-plant poisoning. Even more *CSP* genes are turned on upon abamectin insecticide exposure (Figure 5). Under severe toxic chemical stress conditions, a drastic up-regulation of a *CSP* chainwork is observed in the tissues involved in lipid metabolism and xenobiotic degradation, i.e., gut, fat body and epidermis (Figure 5). 

Interestingly, among these twenty *BmorCSP* genes, only one (*BmorCSP6*) shows decreased gene expression (down-regulation) over insecticide exposure in many various tissues such as the antennae, the pheromone gland, legs and wings as well as epidermis (see Figure 5) [14]. This may suggest that BmorCSP6 has a very different function than the other BmorCSPs. The expression of *BmorCSP6* in bacteria and in many various insect tissues from epidermis to pheromone gland strongly argues that olfaction and pheromone production are not BmorCSP6 primarily functions. Most surprising and interesting fact is that, alike BmorCSP2, BmorCSP6 exists identically in insects and bacteria [30]. The function of these highly conserved protein sequences is unknown. They may play a conserved role in mechanosensing of droplets or surfaces, i.e., the process that often uses obstruction of flagellum rotation to trigger adhesion, surface-associated movement, biofilm formation and/or bacterial virulence [54,55]. This could explain their contribution to insect epidermal cells, glands, neurons and bacteria. So it could be that one chainwork of *CSPs* is turned off upon exposure to insecticide or bactericide, while another *CSP* chainwork is activated to degrade or expel the infectious toxic agent in a sex or strain-specific manner (Figure 5) [14].

## 4. CSPs for Lipid- and Fatty Acid- Mediated Pathways

Also interestingly, numerous *CSP* genes are used in the nervous system of the silkworm moth, *B. mori*. Under normal conditions, nearly all *CSPs* are expressed not only in the head, but also in moth peripheral organs such as the antennae, the wings and the legs (Figure 5) [14]. 

A similar observation was made by Liu et al. (2016) related to this finding, *CSP* expression throughout the whole body, but mainly in the head and the peripheral organs [15]. With this study, we give two main points for assessment of CSP function in insects: (1) *CSP* is up-regulated by insecticide, and (2) the protein binds specifically to linoleic acid, strongly arguing for a role in fatty acid- and lipid-mediated pathways for adaptation, signaling and immune defense [15]. Most importantly for our present analysis of function is that annotated ESTs in the honeybee show enriched expression of *CSP* in the head, particularly in the brain (see Table S3), strongly suggesting that CSPs have complex actions not only in the insect immune system, but also in the central nervous system and virtually all of the body’s organs, including antennae, wings and legs. 

**Figure 6:**
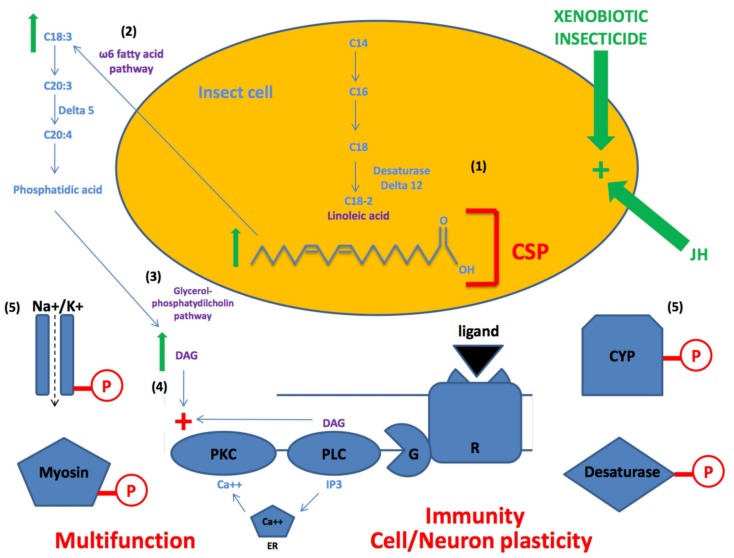
Conjectural model of the role of CSP in activation of linoleic acid/omega6 fatty acid-diacylglycerol pathway upon xenobiotic insecticide or juvenile hormone exposure. (**1**) Some insect cells have the ability to synthesize linoleic acid (C18:2) *de novo* using fourteen-eighteen carbons-fatty acids (C14–C18) and specific desaturases. (**2**) C18:2 is fuel molecule for omega6 fatty acid pathways. Molecules such as arachidonyl-CoA ((Z5,Z8,Z11,Z14)-Icosatetraenoyl-CoA or C20:4) are products of a ∆5 desaturase reaction from eicosatrienoyl-CoA (C20:3) as a direct substrate. (**3**) Synthesis of these fatty acid metabolites leads to phosphatidic acid and therefore to the formation of diacylglycerol (DAG) through the biosynthetic pathway of glycerol-phosphatydilcholins. (**4**) DAG is a relay molecule in intracellular cascades activated by the binding of regulatory chemical ligand (labelled by a black triangle) to G-protein coupled receptor. This triggers the formation of inositol 1,4,5-triphosphate (IP3) and DAG by PLC (phospholipase C). In turn, IP3 releases the calcium ions (Ca^++^) from intracellular stocks in the endoplasmic reticulum (ER). (**5**) DAG (with Ca^++^) activates (**+**) protein kinase C (PKC), which in turn induces specific cellular responses by phosporylating a particular set of cellular proteins (ion channels, myosin, cytochrome P450, desaturase enzymes, etc.). Applying xenobiotic insecticide and/or juvenile hormone (JH) activates (**+**) the DAG pathway and thereby protein phosphorylation (red symbol P) via increased concentrations of C18 and C20 fatty acids on cell growth performance and/or immune response of various tissues, organs and organ systems. The green arrow means that the concentration of C18:2, C18:3 and DAG increases with increasing concentration of xenobiotic insecticides and/or JH. The central role for CSP in ∆12-fatty acid pathway associated with transport of C18:2 for multifunction, immunity, cell development, tissue growth and neuronal plasticity is shown in red.

Based on these results, we propose that CSPs play a key role in activating the omega6 fatty acid pathway, which is necessary to produce diacylglycerol (DAG) that will in turn activate phospholipase kinase C and phosphorylation of many various different proteins (Figure 6) [56]. DAG-mediated protein phosphorylation is an essential requirement not only of neuron depolarization/repolarization (sodium/potassium channels), signal transduction (transmembrane receptor) and/or muscle contraction (myosin motor protein), but also of the activation of lipid biosynthetic and degradative enzymes such as cytochrome oxidases (CYPs) and delta (∆)-desaturases (Figure 6). These enzymes both regulated by phosphorylation/dephosphorylation processes are essential for xenobiotic degradation, storage of fatty acids and pheromone production [57,58,59,60,61,62]. Accordingly, they represent very important molecular elements for organismal adaptation and evolution.

In insects, it has been shown that the DAG-phospholipase C (PLC) pathway can be regulated by juvenile hormone (JH) [63], while exposure to pyrethroid insecticide can stimulate protein phosphorylation activity in the brain of mammals [64]. This suggests that insecticide exposure can stimulate the DAG-PLC pathway in many various tissues of the insect body, not only by a stimulatory effect on the production of C18-linoleic acid (LA) and lipid omega6 fatty acids production, but also via an indirect effect on JH release [65,66,67]. A plethora of pleiotropy across CSPs and binding protein families seems to be necessary to recognize a multitude of targets and phosphorylation sites in a huge variety of complex cell-cell and intracellular signaling pathways in many diverse organisms. 

Interestingly, microbes are known to produce LA and to carry a gene related to JH [68,69]. Both LA and JH are known to be crucial for many cell functions in worms and arthropods, particularly in growth, developmental, reproductive and innate immune systems [70,71,72]. In insects, JH is a sesquiterpenoid hormone produced by the *corpora allata* and is present throughout nymphal, larval and adult life. Most insect species produce only one JH-type (type III), but only butterflies (and moths) produce other JH types such as JH-0, JH-I and JH-II. Flies produce the form JHB3 (JH-III bisepoxyde) [73]. For LA, it has long been a debate about synthesis of C18:2^∆9,12^ lipids in insects as most species were thought to be lacking ∆12 desaturases, the enzymes capable of inserting a double bound at the ∆12-position. In fact, it is clear now that insects such as ants, bees, cockroaches (*Periplaneta*), crickets, moths, termites and wasps can synthesize LA *de novo* [74]. It is well known that oleic acid and LA are “necromones”, pheromones given off by a dead organism as described in ants, bees, cockroaches and crickets [75]. Oleic acid is also known as a main precursor molecule of LA and male sex pheromone in the parasitoid wasp *N. vitripennis* [76]. Similarly, LA is a precursor of sex pheromone compound in moth species such as Bombycidae, Crambidae and arctiid moths [77,78,79]. Finally, LA and C18 fatty acids are known to play a key role in moth development as demonstrated in the crambidae species, *Ostrinia nubilalis* [80]. Therefore, the interactions of CSPs and LA/linolenic acid on fatty acid pathways would be crucial to regulate many various physiological systems, including pheromone biosynthesis, growth, development, tissue regeneration and/or toxin/insecticide immune responses in many various species from bacteria to all various groups of insects. 

Accordingly, an adequate number of CSPs may be important to interact with the various intermediary molecules of the LA/fatty acid pathway. EFN87902/EFN72587, NV16109 and AmelGB10389 would have the function to transport LA in hymenoptera because they clearly group together with BtabCSP1 in our evolutionary analysis of insect CSPs (see Figure 3). The bee protein AmelASP3c is highly expressed in the antennae (in sensilla trichodea B and sensilla basiconica), but it is absolutely not restricted to antennal sensilla. It is also found on wings and legs, suggesting a very much more general function than queen pheromone recognition for this CSP protein [81,82]. The tissue distribution of CSP-EST sequences in the honeybee *A. mellifera* confirms that *ASP3c* is not specifically expressed in a peripheral organ such as the antennae. We find that the part of the insect body that expressed *ASP3c* gene the most is the head, and the main organ for ASP3c is the brain (Table S3). So, we propose that ASP3c transports fatty acid lipids instead of pheromones for instance for process, growth, development and/or regeneration in neurons and other cell types harbored by the central nervous system in the honeybee. 

Similarly, in situ hybridization to RNA in antennal tissue section from *D. melanogaster* shows that CSP (DmelOSD) associates with the sacculus (involved in hygrosensing) and patches of sensilla coeloconica distributed on various parts of the fly antennae [83,84]. DmelOSD homologs are also found in the hemolymph in response to microbial inoculation [46]. Therefore, it is clearly shown that many various types of sensilla and tissues as well as the circulatory fluid bathing these tissues possess the same CSPs. Such expression profiling in Hymenoptera and Diptera is in agreement with a role of CSPs in the transport of lipids, which are essential for cell survival and adaptation. The gene *CG9358* (related to *AmelGB13325* and *NV16108*) is under the control of embryo and tissue developmental factors and governed by transcription factors involved in circadian rythms [85,86,87]. So, it is worth noting that flies that express only four *CSPs* cannot synthesize LA de novo and therefore require a dietary resource of C18:2 lipid [12,37,88]. On the basis of numerous observations, we propose that the inability of flies to synthesize LA and LA derivatives is caused by lack of specific enzymes, i.e., ∆12 desaturases, and a specific group of small transport proteins of the CSP family (Group V, see Figure 3), as an example of the importance of *CSPs* in cell evolution.

## 5. Genetic Editing of CSPs for Insecticide Resistance 

Interestingly, it is also worth noting that the *CSP* family is subjected to RNA editing and that this RNA editing may significantly increase expression and activity of the protein (see Figure 1) [13,16,17,19,31]. Edited versions or mutations are not limited to A-to-I and/or C-to-U conversion, but protein diversity and multifunction in CSPs seems to be brought on by many other mechanisms, including insertion of specific amino acid motifs and residues at the protein level [13,16,17,19,31] (see Figure 1). Insertion of amino acids such as Glycine near Cysteine at key position on the protein structure may modify the profiling of alpha-helices, which are essential components of the protein-fatty acid lipid interaction [7,8,9,10,11,13,16,17,19,20]. The data obtained in moths corroborate the hypothesis that RNA editing compensates for a small genome size and/or for the decreased diversity of *CSP* genes, as reported here for honeybees and parasitoid wasps. RNA editing in *CSPs* seems to be crucial for lipid transport and thereby cell type diversity. 

In these insect species such as lice, bees and wasps with only six to eight *CSPs*, only RNA and/or protein editing mechanisms could allow them to use CSPs for the transport of a high diversity of lipid-ligands as proposed for moths [13,14,15,16,17,30,31] (see Figure 1,Figure 2,Figure 3,Figure 4,Figure 5,Figure 6). In beetles, a huge amount of RNA variants are found for *AAJJ0012I* and *AAJJ0283B* [38]. In locusts, a high number of copies of genes (> fifty) are used for differential expression pattern of *CSPs* in relation with phase change [89,90]. However, some of the RNA clones identified in *Locusta migratoria* adults indicated the presence of subtle nucleotide replacements (A-to-G, A-to-C, C-to-A and U deletion) between some specific *CSP* sequences, suggesting the occurrence of RNA editing in *CSPs* not only in moths and beetles, but also in locusts and grasshoppers in the Acrididae family [4]. The American cockroach (*P. americana*) shows numerous variant N-terminal sequences for CSP proteins, similarly to *Bombyx, Tribolium* and *Locusta*. So, this genetic regulation or plasticity of *CSPs* through RNA editing also occurs in the order Blattodea [2,5]. Mutation is also described in whiteflies where *CSP* mutations appear to be biotype-specific [15]. Many mutant peptide fragments sequenced in *Bombyx* are very similar to CSPs from Hemipteran or Dipteran species, strongly suggesting that RNA editing of *CSP* is important for many insect species [13,20]. 

Here, we report about the importance of RNA editing in *CSPs* from groups of social insects such as the honeybee. Analyzing EST sequences from GenBank [91], we find a high number of mutations (RNA editing) in the order Hymenoptera, particularly in the bee brain (Table S3).

Analyzing nucleotide sequences encoding CSP in *A. mellifera* using blastn algorithm for GenBank EST sequences in FlyBase shows numerous subtle nucleotide switches such as A-to-G and U-to-C at least for three *CSPs* from the honeybee: *AmelASP3c*, *GB17875* and *GB19453* (Table S3). Such a high number of mutations in the bee brain suggest the importance of RNA editing in *CSPs* for the insect central nervous system (Table S3). Editing of *CSPs* (A-to-G, U-to-C, C deletion, U-to-G, G-to-U, U-to-A, A-to-C, A-to-G, G-to-C and C-to-G) in the bee brain (Table S3) may be linked to task performance and social behavior [92,93].

Subsequently, we propose that RNA editing as well as rapid evolution and positive selection in *CSP* duplicates (increasing the number of *CSP* genes) has largely contributed to the development of new protein functions, resulting in high insecticide resistance capacities, for instance, particularly in insect species such as beetles, moths and whiteflies [13,14,15,38]. The beetle has developed resistance to more than fifty different chemicals belonging to all major insecticide chemicals [94,95]. Correlatively, it expresses a number of about nineteen *CSP* genes [38]. Moth larvae that can develop very fast insecticide resistance even to new chemicals retain about twenty *CSP* genes as described in the silkworm *B. mori* [14,96,97]. In the whitefly *B. tabaci*, some biotype-specific variations exist within *CSPs*, which could underlie such a high insecticide resistance capacity observed in whiteflies of Q-biotype, in particular for neonicotinoid molecules [15,98,99]. So not only detoxification genes, but also the number of *CSPs* and/or *CSP*-RNA variants as well as their ability to bind to specific lipids, LA and other fatty acids, as well as xenobiotic compounds, may be crucial for diverse insect species, strains or biotypes to develop a high resistance capacity to chemical insecticide molecules.

Insect CSPs are crucial in chemical communication by recognizing environmental chemical stressors [14,15]. The RNA editing of *CSP* genes is not the only way to be involved in insecticide resistance in insects [13,16,17]. Besides expression and mutation, there are examples of multifunction to explain how *CSPs* may play a central role in insecticide resistance. In moths, *CSPs* respond to avermectins [14]. In whiteflies, CSPs are lipid carriers and xenobiotic transfer proteins [15]. All RNA editing in *CSPs* may be associated with insecticide resistance for the transport of many different types of ligands and/or activation of a variety of degrading enzymes such as cytochromes P450 (CYPs) and JH esterases [14,15]. *Bombyx* CSP-RNA is characterized by high mutation rates in many various sensory and non-sensory tissues, including the pheromone gland [13,16,17]. In the flour beetle *T. castaneum*, CSP-RNA variants are mainly found in the hindgut and Malpighian tubules in response to insecticide [38]. RNA editing is also crucial to mediate insecticide (ivermectin) resistance through specific point mutations in GABA receptors (resistant to dieldrin) [100]. Similarly, RNA editing in sodium channel in mosquito plays a role in pyrethroid resistance [101], however, another study showed that RNA and genomic DNA sequences from the same *Aedes aegypti* individual did not support the involvement of RNA editing in permethrin resistance [102]. Therefore, the importance of RNA editing in insecticide resistance may depend on chemical families, insecticide structures and insecticidal properties or modes of action. RNA editing in *CSPs* is an extremely important component of resistance to avermectins known to block the transmission of electrical signals in insect nerve and muscle cells by targeting glutamate-gated chloride channels, and neonicotinods, which target nicotinic acetylcholine receptors (nAChRs) [13,14,15,16,17]. More work needs to be performed to check whether all RNA editing in mechanisms and molecular targets of avermectin and neonicotinoid pesticides are associated with insecticide resistance. 

## 6. Genetic Plasticity of *CSPs* for Neuroplasticity

So, the bees being so depauperate genetically, lacking many detoxification enzymes and CSPs, become very sensitive to the toxicity of many various foreign chemicals [103]. This does not, however, exclude the possibility that other CSPs or groups of CSPs may be essential for the bees to accomplish special feats such as odor memorization and specific social behavior. The high expression levels of CSPs and CSP variants that we have detected in the nervous system of the honeybee, particularly in the brain (see Table S3), suggest a possible role of CSPs in learning and memorization processes. A possible role of CSPs in neuroplasticity is also suggested by gene knockout experiments. *CSP* (*AmelGB10389* on LG1 chromosome) has been knocked out in bees, resulting in an archaic development of the brain [104]. 

When a cell differentiates or acquires a defined specialized function, it is supposed to undertake major changes in its size, shape, protein synthesis, metabolic activity, and overall function which at the end will serve a defined tissue or organ. Despite their different shapes, colors and functions, all various tissues or organs, even in most complex multi-cellular organisms such as humans, come from the same basic common totipotent cell, i.e., an immature stem cell capable of giving rise to any cell type from an embryo or an undifferentiated cell that can renew itself and can differentiate to provide any specialized cells types of a given tissue or organ in an organism at a certain point in time [105,106,107,108]. Our finding in insects indicating such a huge diversity in base mutations and protein changes at the level of CSPs may bring an answer not only about the high capacity of insects for chemical resistance, but also about the basis for the development of stem cells as well as for structural and functional reactions of neurons. 

Neuronal plasticity is reported in arthropods and insects as various responses involving any change in the brain from dendrite regeneration, axon sprouting and synapse formation, resulting in specific behavioral adaptations [109]. It could be that the multi-function of CSPs in carrying all sorts of lipids and adhering to all sorts of surfaces plays a key role in the mushroom body neuropiles, i.e., in olfactory learning and memorization processes, in particular in adult social insects such as the honeybee and long-lived migrant species of moths such as the black cutworm moth *Agrotis ipsilon* [110]. In long-lived species of moths, it has been shown that JH known to exert pleiotropic functions during the whole insect life cycle, controlling many various physiological systems from metamorphosis, tissue development and pheromone activities, is also essential for peripheral and central nervous processing of sex pheromone and/or plant odor [110,111,112,113]. Pleiotropic proteins such as CSPs and pleiotropic hormones such as JH may interact with each other to govern the switches observed in the brain responses to odorant signals. Controlling LA pathways (see Figure 6), both CSP and JH may allow differential processing of pheromone and plant odor, i.e., activation or transient blockade of specific integrative centers in the brain [112]. This needs to be elucidated by searching for the ability of CSPs to interact with JH and/or to locate precisely the site of CSP expression not only in the brain structure, but also in the bee or moth neuron. 

Expression of *CSPs* in the neural system of insects to control DAG and protein phosphorylation may be an example of neofunctionalization of this protein gene family for neuroplasticity, neurogenesis, synaptogenesis, the formation of new synapses and generation of new neuron connections (see Figure 6). So far, we can only discuss abundant pleiotropy in CSPs for insect defense and lipid metabolism [13,14,15,16,17,18,19]. Pleiotropy (functional plasticity or multi-function) of CSPs is demonstrated by the study of Liu et al. in the whitefly *B. tabaci*, where CSP1 is involved in the response against thiametoxam by interacting with LA, while two other CSPs rather involve in the transport of bark plant phenolic chemicals such as cinnamaldehyde and derivatives [15]. Cinnamaldehyde and cinnamon leaf oil are known to retain the ability to kill bacteria, fungi, mosquito larvae and many insects on contact as well as to act as a strong repellent long afterwards [114]. Therefore, cinnamon oil seems to represent a very ancient system that plants have developed for defense against insects and microbial pathogens. Plants have been interacting with herbivorous insects and bacterial fauna for hundreds of millions of years. In turn, herbivorous insects (and bacteria) have certainly developed their own defense system to counteract the panoply of poisonous chemicals released by the plant for My(Million years). Liu et al. have demonstrated that CSPs are essential for cell defense through the binding of lipids and xenobiotics [15]. Therefore, lipid transport and the sequestration of toxic xenobiotic chemicals may represent some ancestral CSP functions, i.e., used by bacteria and early eukaryotes to grow and adapt to the natural environment. Later, new CSP functions may have been crucial for the appearance and development of the nervous system, including not only the formation of many types of brain cells, but also neural plasticity.

A lack of variation for genetic plasticity, RNA editing and/or protein recoding would have led to a lack of evolutionary perspective for adaptive capacity in all diverse organismal associations such as the moth and the green plant or the bee and the flower. The complex system of pleiotropic genes such as *CSPs* enrolled not only in lipid and FA biosynthesis, incorporation, transport and metabolism, but also in immunity, tissue growth and neuroplasticity is certainly a big part of the most ancient evolutionary components of the cellular system of living organisms in an environment that is constantly changing. 

## 7. Conclusions and Future Research 

In this review, we do not give justice to the eluding nature of the CSP protein family, but address all the known aspects of this protein gene family: the post-transcriptional modification of the genes encoding chemosensory proteins, the genomic organization of *CSPs* as described here in honeybee and jewel wasp, the phylogenetic distribution of CSP sequences from insects/arthropods and bacteria, their gene expression profiling and tissue-distribution, and their multifunctionality, as well as their role in lipid fatty acid pathways for various physiological systems, including mainly insect defense and insecticide resistance. Then, we attempt to engage in an understanding of their neofunctionalization or ability to interact with lipids and fatty acids for neuroplasticity. 

Although the biochemical mechanisms of CSPs in the resistance against insecticide has not been fully investigated, a role of CSPs at different levels of the insect immunological defense is strongly supported by the ability of whitefly CSP1s to interact with lipids, while whitefly CSP2s and CSP3s have the ability to interact directly with specific xenobiotic compounds such as cinnamaldehydes from plant oils [15]. 

An issue for debate about a common role of the CSP workchain for neuroplasticity is that most CSPs in the silkworm are enrolled in the nervous system upon normal conditions [14], and that most CSPs in the honeybee are expressed in the brain (this study). It remains to be found if they are involved in the same process from bacteria to insects, if they all have the same function that many organisms from bacteria to insects use, or if some of these CSPs were subjected to specific mutations and acquired a more specialized new function, prior to the birth and development of neuronal cells and/or specific behavioral traits, including those of social insects such as the honey bees and those of migrant species such as the black cutworms. 

In these species, research should be made for RNA/protein mutation on a specific tissue such as the brain and analysis of functional properties, particularly in the insect neuropile where a dense network of nerve fibers, their branches and synapses, together with glial filaments rebuild and reorganize specific synaptic connections, especially in response to learning, memorization and/or brain tissue injury. Pleiotropic CSPs capable of carrying fuel molecules such as lipids and fatty acids might be crucial in these processes of development and neural tissue regeneration. 

In addition, deeper research should concern CSPs, immune cells and/or cells exposed to a panoply of antigenic substances. Human thymus or insect hemocytes can express a prominent diversity of proteins and protein variants in response to infection or environmental contamination, and yet adapts to new conditions and sustains development as well as natural evolution. Apparently, considering the genetic plasticity, RNA editing and true functional pleiotropy characterizing this gene family, *CSPs* could potentially bring an answer for stem cell research, phenotypic evolution and critical thinking in questions of neuroscience. Firstly, because all cells in the body, beginning with the fertilized egg, contain the same DNA, how do the different cell types come to be so different and different enough to yield such a high diversity of tissues or organs, each characterized by a specific specialized function? Secondly, how can the insect brain switch on/off its responses to specific odor signals depending on the environment?

Far beyond the DNA structure, it is well established now that post-transcriptional events such as alternative splicing and RNA editing are able to subtly modify proteins to diversify their structures for multi-function. In particular, new mechanisms to be found in the expression of CSPs may serve to explain neuroplasticity in the nervous system, the diversity of cellular responses in the immune system, and fate as well as transformation in the stem cells of a newborn organism [19,31,115]. In this review of multiple genetic events, using *CSPs* as a model study, we discuss how RNA editing and activation of lipid fatty acid pathways can contribute to specific innate and adaptive immune responses and/or to specific neurobiological development (brain–immune interactions) in parasitoid wasps and social insects such as the honey bees. RNA editing is probably required to circumvent a rather limited repertoire of *CSP* genes as found for parasitoids and bees. We find only six and eight *CSP* genes in the honeybee *A. mellifera* and the solitary parasitoid emerald jewel wasp *N. vitripennis*, respectively. Gene structure and intron boundary show gene duplication, but our phylogenetic tree analysis shows a distinct evolutionary route between honeybee and pteromalid parasitoid wasp *CSPs*. We report here that a particular group of “ancient” *CSPs* is closely related to metabolic *CSPs* from bacteria and aquatic species of arthropods, perhaps suggesting that *CSPs* are the products of a duplication that took place Bya in the most ancient (Archaeal) organismal lineage and it is very likely that this duplication happened to be crucial for the adaptation of Archaeal cells. 

Further duplications might have happened to promote adaptation and evolution of prokaryote and eukaryote cells in diverse environments [116]. Therefore, genetic plasticity (gene duplication and RNA editing) in *CSPs* should be investigated not only in the neural stem cells of the insect brain, but probably also in the filamentous bacterial cells to uncover cell–cell adhesion and interaction mechanisms. This would be an essential prerequisite to understand neuroplasticity, tissue differentiation, organ development, cell proliferation, bacterial infection, virulence and immune defense. Genetic editing or RNA plasticity in *CSPs* is a very new and promising subject to allow for a better understanding of the role of small soluble binding protein carriers in insects and bacteria to be explored by both entomological and medical healthcare industries and, most likely, of evolutionary processes that gave rise to life diversity at every level of biological organization [117]. 

## Supplementary Materials

The following are available online at https://www.mdpi.com/2073-4425/11/4/413/s1, Figure S1: Genomic organization of *B. mori* and *T. molitor CSPs* [14,41]. The red arrow shows the position of *double-intron* genes (outside the main group of *CSPs*). Table S1: *CSP* genes identified by in silico analaysis of honeybee *A. mellifera* database. Table S2: CS*P* genes identified by in silico analaysis of jewel wasp *N. vitripennis* database. Table S3: Tissue distribution of CSP-EST sequences in honeybee *Apis mellifera* (*carnica*).
