# Supplementary material for: Comprehensive History of CSP Genes: Evolution, Phylogenetic Distribution and Functions
Source: Genes (Basel). 2020 Apr 10;11(4):413. doi: 10.3390/genes11040413 (PMC7230875; doi:10.3390/genes11040413)
Supplement: Supplementary file 1 [file genes-11-00413-s001.zip › Liuetal.2020SupMat/Liuetal.GENES2020TableS1.docx]

**Table S1.** CSP genes identified by in silico analaysis of honeybee *A. mellifera* database (BeeBase, <http://www.genome.gov>, <http://www.racerx00.tamu.edu>). Access numbers in italic refer to genomic DNA sequences (*Scaffold number). Int1-int2: introns 1 & 2 from AmelGB19453 (Chromosome LG2).

| Name | **Genome Size (Mb)** | **Gene Size (bps)** | **Intron Size (bps)** | **Access Numbers**  **(NCBI)** |
| --- | --- | --- | --- | --- |
| ***Apis mellifera*** | 264 |  |  |  |
| GB13325 |  | 1866 | 1488 | *AADG05008379**, NW_001253742, NM_001077819, XM_001120200, AJ973402, DQ855487,  ENSAPM0988/24007/35375/28441, S.C_Grp1.373000005B/7A, NP_001071287,  XP_001120200, CAJ01449, ABH88174, *LG1-NC_037638*, *QIUM02000010* |
| GB10389 |  | 1251 | 936 | *AADG05006819**, NM_001078661, DQ855486, S.C_Grp1.373000004B/6A, NP_001072129,  ABH88173, *LOC751767, GB43823, LG1-NC_037638*, *QIUM02000010* |
| GB19453 |  | 651 | 3570 (int1)  297 (int 2) | *AADG05008379**, NC_007071, NW_001253275, NM_001077810, AJ973398, DQ855483,  S.C_Grp2.2000002B, NP_001071278, XP_392197, ENSAPM13974,  CAJ01445, ABH88170, *GB55547*, *LG2-NC_037639, QIUM02000012* |
| ASP-3c |  | 4221 | 900 | *AADG05001880**, NC_007074, NW_001253364, NM_001011583, AF481963, AJ973401, DQ855484,  S.C_Grp5.4000024B/81A, NP_001011583, AAN59784, ENSAPM16534, CAJ01448,  ABH88171, *GB52324*, *LG5-NC_037642*, *QIUM02000016* |
| GB19242 |  | 880 | 493 | *AADG05000792**, NC_007074, NW_001253364, NM_001077814, XM_379006, AJ973400, DQ855485,  S.C_Grp5.4000026B/83A, NP_001071282, XP_397006, ENSAPM16535/25203/23915/18745/22653, CAJ01447, ABH88172, *GB52326*, *LG5-NC_037642, QIUM02000016* |
| GB17875 |  | 463 | 112 | *AADG05001880**, NC_007077, NW_001253516, NM_001077820, XM_001120077, XM_001120077, AJ973399,  DQ855482, S.C_Grp1.236000007B/7A, NP_001071288, XP_392945, ENSAPM17207,  CAJ01446, ABH88169, *GB43823*, *LG8-NC_037645*, *QIUM02000032* |
