# Supplementary material for: Comprehensive History of CSP Genes: Evolution, Phylogenetic Distribution and Functions
Source: Genes (Basel). 2020 Apr 10;11(4):413. doi: 10.3390/genes11040413 (PMC7230875; doi:10.3390/genes11040413)
Supplement: Supplementary file 1 [file genes-11-00413-s001.zip › Liuetal.2020SupMat/Liuetal.GENES2020TableS2.docx]

**Table S2.** CSP genes identified by in silico analaysis of jewel wasp *N. vitripennis* database (NasoniaBase, http://hymenopteragenome.org) Access numbers in italic refer to genomic DNA sequences (*: Scaffold number). Int1-int2: introns 1 & 2 from NV16079 (chromosome 4).

| Name | **Genome Size (Mb)** | **Gene Size (bps)** | **Intron Size**  **(bps)** | **Access Numbers**  **(NCBI)** |
| --- | --- | --- | --- | --- |
| *Nasonia vitripennis* | 330 |  |  |  |
| NV16075 |  | 530 | 140 | AAZX01001008*, XM_001600061, XP_001600111, LOC100115372 |
| NV16076 |  | 548 | 307 | *AAZX01001008**, XM_001601477, XP_001601497, *LOC100113667* |
| NV16077 |  | 467 | 80 | *AAZX01001008**, XM_001600139, XP_001601446, *LOC100113624* |
| NV16078 |  | 576 | 174 | *AAZX01001008*,* GE387616, GE387770, *unannotated* |
| NV16079 |  | 3126 | 1596 (int1)  981 (int2) | *AAZX01001008**, XM_001600138, XP_001600188, *LOC100115474* |
| NV16080 |  | 739 | 322 | *AAZX01001008**, XM_001601583, XP_001601633, *LOC100113725* |
| NV16108 |  | 1628 | 1256 | *AAZX01001011**, XM_001603872, XP_001603922*, *LOC100113847* |
| NV16109 |  | 1675 | 1354 | *AAZX01010911**, XM_001601035, XP_001601085, *LOC100116643* |
