# Supplementary material for: Comprehensive History of CSP Genes: Evolution, Phylogenetic Distribution and Functions
Source: Genes (Basel). 2020 Apr 10;11(4):413. doi: 10.3390/genes11040413 (PMC7230875; doi:10.3390/genes11040413)
Supplement: Supplementary file 1 [file genes-11-00413-s001.zip › Liuetal.2020SupMat/Liuetal.Genes2020TableS3-CORR.docx]

# Table S3 Tissue distribution and mutation of CSP-EST sequences in honeybee *Apis mellifera* (*carnica*). *ASP3*/Score: 355.334-720.087, E value: 1.40852e-96-0; *GB10389*/ - (undetected)*; GB13325*/Score: 468.328-702.246, E value: 3.1732e-131-0; *GB17875*/Score: 680.44-688.37, E value: 0; *GB19242*/ - (undetected)*; GB19453*/Score: 385.07-702.246, E value: 3.4273e-106-0. Base mutations are indicated in brackets. EST sequences (access numbers) are from FlyBase (flybase.org): bee female antennae, head, brain normalized, whole body 2 days old, whole body 2 days old and 3 days old, and whole body 5 days old *A. mellifera* cDNA clones (honeybee cDNA collection/Riken).

| **Gene** | **Antennae** | **Head** | **Brain** | **Whole body**  **2-days-old** | **Whole body**  **2/3-days-old** | **Whole body**  **5-days-old** |
| --- | --- | --- | --- | --- | --- | --- |
| *ASP3* | BE844261  BE844452 | DB733308  DB734551  DB735567  DB742822  DB749206  DB749617  DB751627  DB761266  DB762293  DB769684  DB776070  DB776467 | BI946526  BI510214  BI512492 | HX290058  HX335094 (<C-32, A>G-229) | HX325409 (A>G-13)  HX371382 (A>U-12, A>G-13, U>C-41, A>U-45, A>C-47, A>U-63, A<-65, A>C-86)  HX371844 | HX306763  HX354883 |
| *GB10389* | - | - | - | - | - | - |
| *GB13325* |  |  |  | HX284110  HX332211 | HX326709  HX372543 |  |
| *GB17875* | BE844373 (A>G-228) | DB728321 (A>G-228)  DB730707 (U>C-44)  DB735162 (U>C-44)  DB736594 (A>G-228)  DB737780 (A>G-228)  DB738505 (U>C-44)  DB739135 (A>G-228)  DB741197  DB741607 (A>G-228)  DB741705 (A>G-228)  DB742987 (A>G-228)  DB744083 (A>G-228)  DB745769 (A>G-228)  DB750182 (A>G-228)  DB751273 (A>G-228)  DB754395 (A>G-228)  DB755064 (A>G-228)  DB757208 (U>C-44)  DB766072 (A>G-228)  DB768622 (A>G-228)  DB770935 (A>G-228)  DB773410 (A>G-228)  DB774790 (A>G-228)  DB778184 (A>G-228) |  |  |  |  |
| *GB19242* | - | - | - | - | - | - |
| *GB19453* |  | DB729040  DB730158 (A>G-207)  DB749517 (U>G-315, A>G-321)  DB745126  DB748785  DB756686  DB775660  DB776360 | BI506218  BI510373 | HX288541 (U>G-21, U>G-24, A>G-27, G>U-56, U>A-57, A>G-66, A>G-73, A>C-79, A>G-89, G>C-94, A>G-196)  HX333149 | HX344271 | HX307536 (C>G-128, <C-218, C>G-251, A>C-265, C>G-276, C<-282, C>G-325, C>G-334) |

# Table S3 Tissue distribution and mutation of CSP-EST sequences in honeybee *Apis mellifera* (*carnica*). *ASP3*/Score: 355.334-720.087, E value: 1.40852e-96-0; *GB10389*/ - (undetected)*; GB13325*/Score: 468.328-702.246, E value: 3.1732e-131-0; *GB17875*/Score: 680.44-688.37, E value: 0; *GB19242*/ - (undetected)*; GB19453*/Score: 385.07-702.246, E value: 3.4273e-106-0. Base mutations are indicated in brackets. EST sequences (access numbers) are from FlyBase (flybase.org): bee female antennae, head, brain normalized, whole body 2 days old, whole body 2 days old and 3 days old, and whole body 5 days old *A. mellifera* cDNA clones (honeybee cDNA collection/Riken).
