# Supplementary figures and images for: Comprehensive History of CSP Genes: Evolution, Phylogenetic Distribution and Functions
Source: Genes (Basel). 2020 Apr 10;11(4):413. doi: 10.3390/genes11040413 (PMC7230875; doi:10.3390/genes11040413)

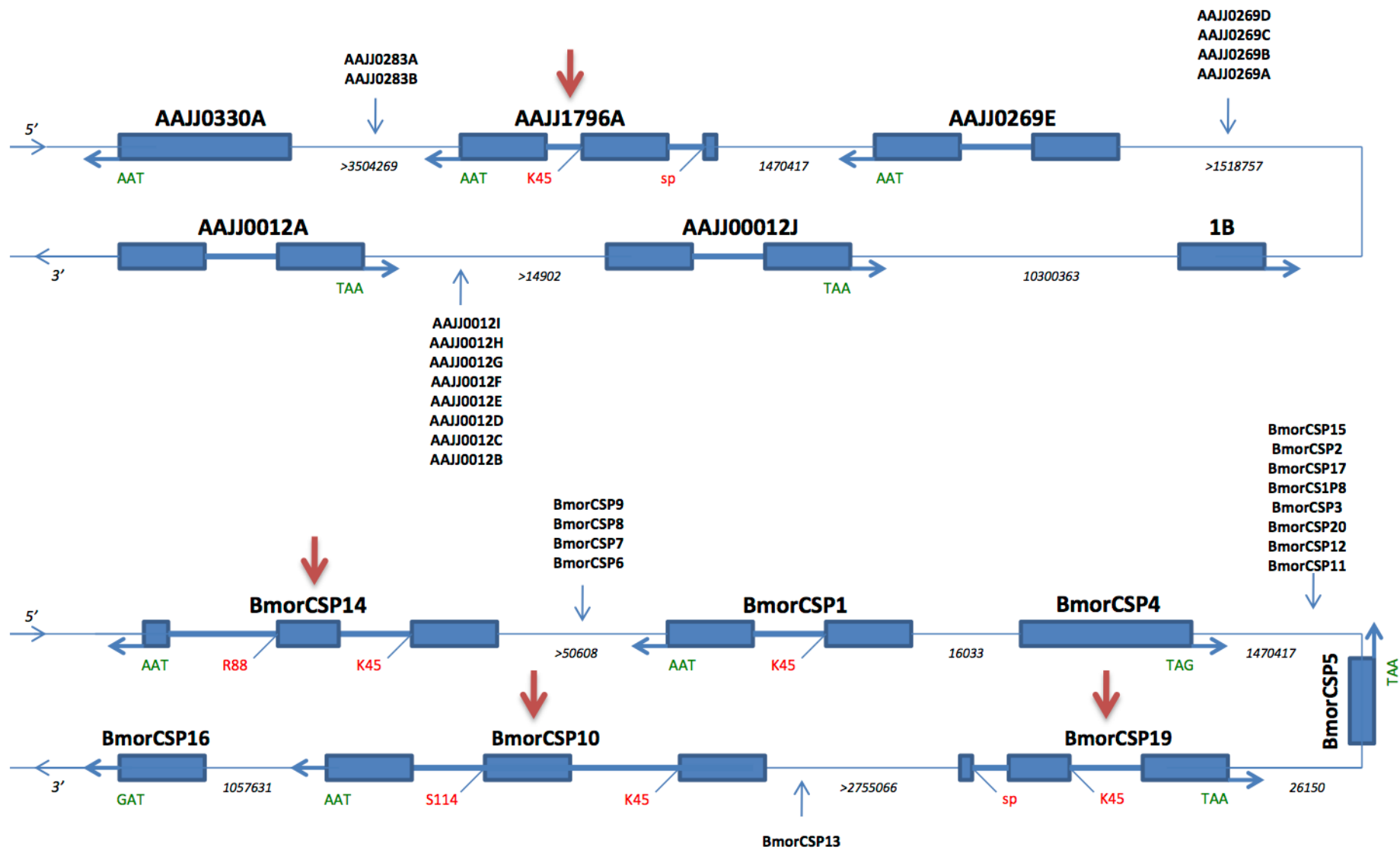

Supplement: Supplementary file 1 [file genes-11-00413-s001.zip › Liuetal.2020SupMat/Liuetal.GENES2020FigureS1.pdf]
